# Identification of novel non-coding small RNAs from *Streptococcus pneumoniae *TIGR4 using high-resolution genome tiling arrays

**DOI:** 10.1186/1471-2164-11-350

**Published:** 2010-06-03

**Authors:** Ranjit Kumar, Pratik Shah, Edwin Swiatlo, Shane C Burgess, Mark L Lawrence, Bindu Nanduri

**Affiliations:** 1 Department of Basic sciences, College of Veterinary Medicine, Mississippi State University, Mississippi State, MS 39762, USA; 2 Institute for Digital Biology, Mississippi State University, Mississippi State, MS 39762, USA; 3 Mississippi Agriculture and Forestry Experiment Station, Mississippi State University, Mississippi State, MS 39762, USA; 4 MSU Life Sciences and Biotechnology Institute, Mississippi State University, Mississippi State, MS 39762, USA; 5 Research Service (151), Veterans Affairs Medical Center, Jackson, MS 39216, USA; 6 Department of Microbiology, University of Mississippi Medical Center, Jackson, MS 39216 USA; 7 Departments of Molecular Biology and Microbiology and Molecular Genetics, Massachusetts General Hospital and Harvard Medical School, Boston, MA 02114, USA

## Abstract

**Background:**

The identification of non-coding transcripts in human, mouse, and *Escherichia coli *has revealed their widespread occurrence and functional importance in both eukaryotic and prokaryotic life. In prokaryotes, studies have shown that non-coding transcripts participate in a broad range of cellular functions like gene regulation, stress and virulence. However, very little is known about non-coding transcripts in *Streptococcus pneumoniae *(pneumococcus), an obligate human respiratory pathogen responsible for significant worldwide morbidity and mortality. Tiling microarrays enable genome wide mRNA profiling as well as identification of novel transcripts at a high-resolution.

**Results:**

Here, we describe a high-resolution transcription map of the *S. pneumoniae *clinical isolate TIGR4 using genomic tiling arrays. Our results indicate that approximately 66% of the genome is expressed under our experimental conditions. We identified a total of 50 non-coding small RNAs (sRNAs) from the intergenic regions, of which 36 had no predicted function. Half of the identified sRNA sequences were found to be unique to *S. pneumoniae *genome. We identified eight overrepresented sequence motifs among sRNA sequences that correspond to sRNAs in different functional categories. Tiling arrays also identified approximately 202 operon structures in the genome.

**Conclusions:**

In summary, the pneumococcal operon structures and novel sRNAs identified in this study enhance our understanding of the complexity and extent of the pneumococcal 'expressed' genome. Furthermore, the results of this study open up new avenues of research for understanding the complex RNA regulatory network governing *S. pneumoniae *physiology and virulence.

## Background

The emerging regulatory roles of RNA in prokaryotic and eukaryotic organisms are expanding the central dogma of molecular biology. While the full spectrum of cellular functions regulated by small non-coding RNA (called sRNA in prokaryotes) are yet to be established, work is going on to identify and study the role of non-coding regulatory RNAs in biological systems. In bacteria alone, more than 150 sRNAs are described [1]. The majority were identified in *E. coli*, and their functional characterization showed that they perform regulatory roles in sugar metabolism [2-4], iron homeostasis [5] and cell surface composition. In bacteria, sRNA also mediates post-transcriptional gene regulation, which can be important in virulence [6,7]. Large-scale identification of sRNAs is a necessary step towards understanding their functions in normal bacterial physiology and virulence.

*S. pneumoniae*, a Gram-positive human pathogen, is the most common cause of community-acquired pneumonia and a leading cause of meningitis, sinusitis, chronic bronchitis, and otitis media [8]. Pneumococci cause approximately 63,000 invasive infections and 6,100 deaths every year in the United States alone [9]. There is a precedent for sRNA involvement in pneumococcal physiology and virulence. Investigation of the CiaRH regulon in *S*. *pneumoniae *strain R6 using classic molecular biology and genetic approaches resulted in the identification of 15 promoters which are regulated by CiaRH, of which five encodes sRNAs [10]. This two component regulatory system CiaRH is involved in maintaining cell integrity, competence and virulence. Expression of these sRNAs was confirmed by northern blots, and analysis of sRNA mutants showed that two of these sRNAs were important for stationary phase autolysis. Two sRNAs identified by experimental approaches in *Streptococcus pneumoniae *strain D39 had demonstrated cis-acting effects on the transcription of adjacent genes [11]. Thus there is a need for increased identification of non-coding functional elements in the pneumococcal genome.

A number of computational as well as experimental approaches have been described for identifying sRNAs in bacteria [12]. Computational methods usually rely upon sRNA conservation in closely related species [12,13] and are often limited to accuracy of transcriptional signal prediction programs (like promoter prediction and rho-independent terminator prediction). Although computational prediction of sRNAs in *S. pneumoniae *TIGR4 using program sRNAPredict2 [14] resulted in a list of 63 sRNAs, only nine were validated by Northern blotting in *S. pneumoniae *D39 strain [15]. This lack of agreement between computational prediction and experimental validation necessitates experimental approaches. Experimental methods for sRNA identification include genetic and molecular biology approaches [6,16,17]. Nowadays, genomic tiling arrays and RNA-seq methods are commonly used for genome-wide transcriptome analysis in bacteria [18]. Expression in the intergenic regions of *E. coli *and *Mycobacterium leprae *were identified using tiling arrays, suggesting the likely expression of small non-coding RNAs [19,20]. A recent study identified 27 sRNAs in *Caulobacter crescentus *using tiling array approach [21]. Using parallel sequencing, a large number of putative sRNAs were reported in *Vibrio cholerae *[22]. Immunoprecipitation with Hfq (sRNA binding protein) antibody followed by deep sequencing identified 64 sRNAs in *Salmonella Typhimurium *[23]. A total of 14 sRNAs identified by molecular biology techniques are described in *S. pneumoniae *(strains R6 and D39). To date, global experimental approaches for sRNA identification in the *Streptococcus pneumoniae *have not been reported. Here we describe a genomic tiling array approach for comprehensive identification of sRNAs in *S*. *pneumoniae *serotype 4 clinical isolate TIGR4. We used whole genome tiling arrays for these analyses because they offer an unbiased view of transcription at the genome level. Another reason was absence of Hfq protein in *S. pneumoniae *which eliminates the possibility of immunoprecipitation based identification of sRNAs.

*S. pneumoniae *TIGR4 genomic tiling arrays identified 50 novel sRNAs in genome, thirteen of which were validated by qRT-PCR. Computational analysis for predicting the function of TIGR4 sRNAs was conducted using Rfam database searches, BLAST searches and sequence motif analysis. Tiling arrays also identified 202 operon structures expressed in TIGR4. Overall, our results provide new insights towards understanding the complex regulatory network of the pneumococcus and underscore the importance of genomic features present in non-coding regions.

## Results

### Transcriptionally active regions in TIGR4 genome

A fundamental aspect unique to tiling array data analysis workflow was defining the baseline for the identification of expressed regions of the genome. Fluorescence intensities of spiked positive and/or negative control probes included in the array design are often used for identifying a probe level threshold for expression. RNA for the tiling array experiment was isolated from *S. pneumoniae *strain TIGR4 [24] during mid-log phase (OD_600 _nm, 0.4-0.5). To derive a baseline for expression in our tiling experiment, we used random probes (~20,000) spotted on the array as negative controls. For positive controls, we utilized *S. pneumoniae *TIGR4 proteome data and selected 35 proteins known to be expressed under identical growth conditions [25]. To minimize sequence based effects on probe intensity, we took adjacent probe intensities into account and applied a pseudomedian filter [26]. The threshold for probe expression was set as 11.0 based on the distribution of the intensities of positive and negative control probes, pseudomedian filter setting, and the accuracy of transcript boundary detection. This threshold intensity had an associated FPR (false positive rate) of 1.63% (Additional file 1). Therefore, probes with intensity values ≥ 11.0 were considered to be expressed.

**Figure 1:**
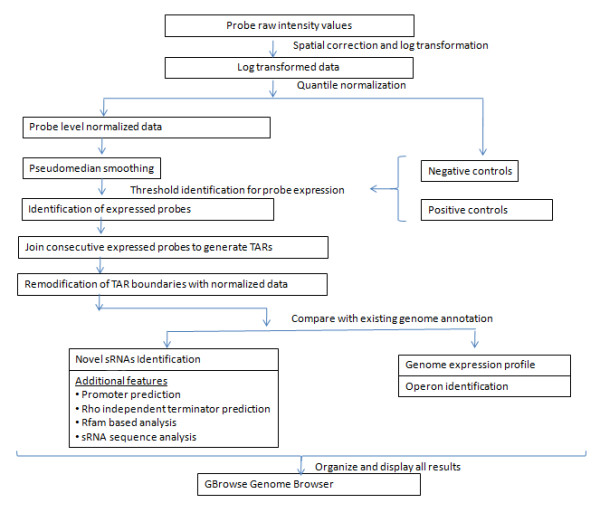
**Tiling array data analysis workflow**. Analysis workflow includes sRNA identification, compilation of sRNA additional features as well as operon identification in *S. pneumoniae *TIGR4.

Consecutive expressed probes were joined together for the generation of transcriptionally active regions (TARs). We identified 2514 TARs in the TIGR4 genome, of which 1324 were found on the nominal forward (+) strand and 1190 were identified on the nominal reverse (-) strand. The genome size of *S. pneumoniae *is 2.2 Mb (2,160,837 bp), of which 88.2% is annotated as genes [24] and rest 11.8% as intergenic region. Overall, our results show that 68% of the annotated regions (that constitutes 50% genes) of the genome are expressed during mid-log phase. In addition, approximately 55% of the intergenic region was expressed which includes sRNAs, UTR regions of mRNAs, and intergenic region within operons. High level of transcription was detected in the repetitive regions present inside the intergenic regions, which were excluded from further analysis. Figure 1, shows the important steps involved in tiling array data analysis.

### Identification and sequence characterization of sRNAs

**Figure 2:**
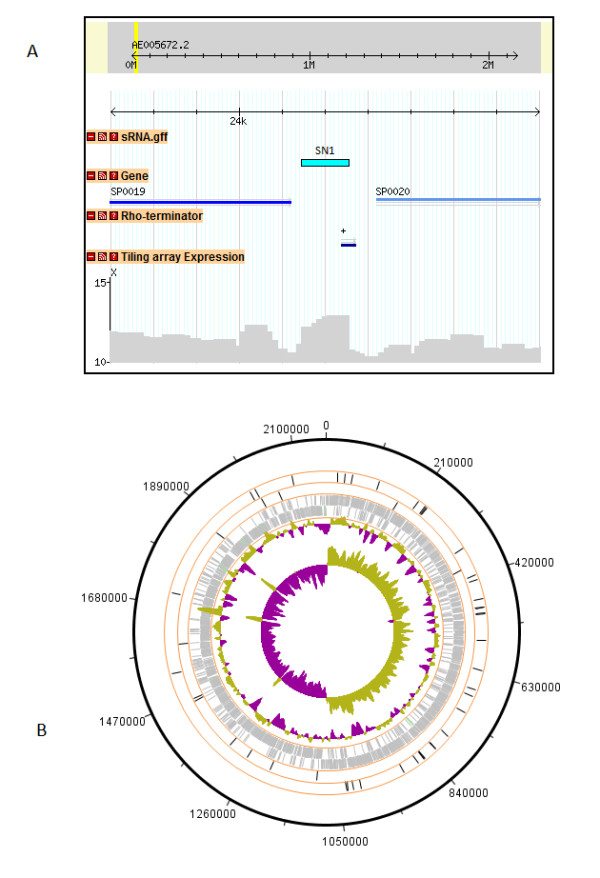
**A *S. pneumoniae *TIGR4 sRNA SN1 visualized in the genome browser**. The sRNA and the additional features are shown as different tracks in the genome browser. sRNA track (in blue) shows the presence of small RNA SN1. Tiling array expression track indicates the higher level of expression in the sRNA SN1 region (located in-between genes SP0019 and SP0020) relative to the intensity threshold cutoff (11.0). Rho-independent terminator track shows a predicted terminator near the 3' end of sRNA. **B. Circular representation of *S. pneumoniae *TIGR4 genome depicting open reading frames and sRNAs**. The outermost track (solid black circle, track one) is TIGR4 genome. With reference to track one, moving inward, tracks two and three represent sRNAs in the forward and reverse strand respectively. Tracks four and five (gray) shows the presence of genes on the forward and reverse strand respectively. Track six is the GC plot and the seventh (innermost track) shows the GC skew of the genome.

Novel non-protein coding sRNAs were identified from the intergenic region of *S. pneumoniae *TIGR4 genome. Our results identified expression in more than 55% of the intergenic region. We excluded intergenic region within operons, small UTRs (untranslated extensions of mRNA) and repetitive regions (including insertion sequences and highly conserved mobile repeat sequences like BOX [27] and RUP [28] elements) from our analysis for identifying novel sRNA. Here we report for the first time, identification of 50 sRNAs (Table 1, sRNA SN1 in Figure 2A) in the genome. The majority of the identified sRNAs were shorter than 200 nucleotides (length range 74 - 480 nucleotides). Since our tiling array design with overlapping probes arranged at 12 bp intervals does not provide a single nucleotide resolution, we cannot accurately identify the exact transcription start/end sited for sRNA. As such the start and end for sRNA in Table 1 refer to the boundaries of transcriptionally active region (putative sRNAs) and in most cases a promoter is predicted within 25 bp of transcript start site (Additional file 2). The overlap between the 50 sRNAs identified in this study and the 63 computationally predicted sRNAs [14] reported for *S. pneumoniae *TIGR4 is very small. Only 8 sRNAs are shared between these two datasets of which four were validated by Northern blotting [11]. A comparison of computationally predicted [14] and experimentally verified sRNAs [10,11] is available (Additional file 3). Five of the sRNAs (SN1, SN5, SN6, SN7 and SN35) were found to be homologs (BLAST identity > 98%, coverage = 100%) of the previously described sRNAs (ccnC, ccnA, ccnB, ccnD and ccnE respectively) from *S. pneumoniae *R6 strain [10]. The identification of all of the five previously identified pneumococcal sRNAs in our study, though not expected *a priori*, nevertheless strengthens our workflow. We utilized these five sRNAs as a benchmark dataset for evaluating the results of our computational analyses of sRNA sequences.

**Table 1:** *S. pneumoniae *TIGR4 sRNAs, their genome location, additional features and comparative genomics.

ID	Start	End	Length (nt)	Strand	Promoter (transcription start site)	Rho independent terminator	Flanking genes Left Right		Rfam prediction	Conservation across other genomes
SN1	24145	24254	110	+	Y	Y	SP0019(+)	SP0020(+)		α β

SN2	40243	40508	266	+	Y	Y	SP0041(+)	SP0042(+)		α

SN3	116167	116372	206	+	Y	-	SP0114(-)	SP0115(+)		α

SN4	171543	171712	170	-	Y	Y	SP0178(-)	SP0179(+)	FMN(Cis-reg,riboswitch)	α β ¥

SN5	228604	228713	110	+	Y	Y	SP0256(+)	SP0257(+)		α β

SN6	230748	230916	171	+	Y	-	SP0257(+)	SP0258(-)		α β

SN7	233177	233262	93	+	Y	Y	SP0260(+)	SP0261(+)		α β

SN8	350572	351050	479	+	Y	Y	SP0372(+)	SP0373(+)	RNaseP_bact_b	α β ¥

SN9	414094	414215	122	+	Y	-	SP0439(+)	SP0440(+)		α

SN10	467128	467294	172	+	Y	-	SP0486(+)	SP0487(-)	FMN(Cis-reg,riboswitch)	α β ¥

SN11	623211	623332	122	+	Y	Y	SP0649(-)	SP0650(-)		α

SN12	667995	668092	98	+	Y	Y	SP0700(-)	SP0701(+)	PyrR(Cis-reg)	α β

SN13	681801	681922	122	+	Y	-	SP0715(+)	SP0716(+)	TPP(Cis-reg,riboswitch)	α β ¥

SN14	783289	783434	146	+	Y	-	SP0834(+)	SP0835(+)		α β

SN15	821508	821581	74	+	Y	-	SP0873(+)	SP0874(-)		α

SN16	821892	822301	410	+	Y	Y	SP0873(+)	SP0874(-)	tmRNA	α β ¥

SN17	853100	853586	487	+	Y	-	SP0897(+)	SP0898(-)		α

SN18	854355	854559	205	+	Y	-	SP0898(-)	SP0899(+)		α

SN19	855530	855627	100	+	Y	-	SP0899(+)	SP0900(-)		α

SN20	869478	869791	318	+	Y	Y	SP0915(-)	SP0916(+)		α β

SN21	1005291	1005532	242	+	Y	Y	SP1068(+)	SP1069(+)	T-box(Cis-reg)	α β

SN22	1033894	1034015	125	+	Y	-	SP1100(+)	SP1101(-)		α

SN23	1324023	1324276	256	-	Y	-	SP1400(-)	SP1401(+)		α

SN24	1529942	1530039	98	+	Y	-	SP1629(+)	SP1630(+)	T-box(Cis-reg)	α

SN25	1592924	1593285	362	-	Y	-	SP1691(-)	SP1692(+)		α

SN26	1989967	1990063	97	+	-	-	SP2078(+)	SP2079(-)		α

SN27	2086051	2086304	277	+	Y	Y	SP2168(+)	SP2169(-)		α

SN28	485360	485540	181	+	Y	-	SP0502(+)	SP0503(-)		α

SN29	497140	497360	221	+	Y	-	SP0516(+)	SP0517(+)		α

SN30	499750	499970	231	+	Y	-	SP0518(+)	SP0519(+)		α β

SN31	2000722	2001113	392	+	Y	-	SP2092(+)	SP2093(+)		α

SN32	1022430	1022539	121	+	Y	-	SP1086(+)	SP1087(+)		α

SN33	392134	392231	105	-	Y	-	SP0411(-)	SP0412(-)		α β

SN34	1706645	1706890	246	+	Y	-	SP1790(+)	Spt11(+)	6S	α β

SN35	209748	209905	158	+	Y	Y	SP0239(+)	SP0240(+)		α β

SN36	423848	423992	145	+	Y	Y	SP0451(+)	SP0452(-)		α

SN37	557778	557971	194	+	Y	Y	SP0587(-)	SP0588(+)		α β

SN38	485578	485759	182	+	Y	-	SP0502(+)	SP0503(-)		α

SN39	721337	721446	110	+	Y	-	SP0761(+)	SP0762(+)		α β

SN40	907168	907301	134	+	Y	Y	SP0958(+)	SP0959(+)	L20_leader(Cis-reg)	α β

SN41	1037030	1037185	166	+	Y	-	SP1104(+)	SP1105(+)	L21_leader(Cis-reg)	α β

SN42	1214232	1214365	137	-	Y	Y	SP1278(-)	SP1279(-)	PyrR(Cis-reg)	α

SN43	1275596	1275742	147	-	Y	Y	SP1355(-)	SP1356(-)	L10_leader(Cis-reg)	α β ¥

SN44	1460966	1461207	242	-	Y	Y	SP1551(-)	SP1552(+)	yybP-ykoY(Cis-reg)	α β

SN45	2005540	2005697	181	-	Y	Y	SP2097(-)	SP2098(-)		α

SN46	2048539	2048648	112	-	Y	-	SP2136(-)	SP2137(+)		α

SN47	56069	56190	122	+	-	-	SP0051(+)	SP0052(+)		α β

SN48	1102915	1103083	169	-	-	-	SP1166(-)	SP1167(-)		α

SN49	1455217	1455362	146	-	Y	-	SP1547(-)	SP1548(-)		α

SN50	1874532	1874844	313	-	-	-	SP1966(-)	SP1967(-)		α β

sRNA sequences conserved in; α-different *Streptococcus pneumoniae *strains like CGSP14, G54, Hungary19A-6, R6, D39. β-different species of *Streptococcus *like *S. mitis, S. gordonii, S. sanguinis SK36*. ¥-other species outside *Streptococcaceae *(for example *Lactobacillus, Clostridium*, and *Bacillus*). The start and end represents the boundaries of identified TAR (transcriptionally active region) which is a potential sRNA region.

The expression of sRNAs showed a strong bias towards the forward strand (38 sRNAs) relative to the reverse strand (12 sRNAs) even though the distribution of protein coding genes in TIGR4 is almost equal for both strands of DNA. We found that the TIGR4 genome has gene orientation bias, a common feature of low-GC (Gram-positive) organisms. Approximately, half of the total genes were located to the right of the origin of replication, of which 79% are transcribed in the same direction as DNA replication and vice versa [24] (Figure 2B). Since two thirds of the identified sRNAs were located to the right of origin of replication, the majority of the sRNAs in our study were expressed in the forward strand.

Transcription is usually facilitated by promoter sequences located in the 5' upstream region on same strand of DNA. Earlier comparative genomics studies also have reported the presence of rho-independent transcription terminators as evidence for the identification of sRNA [29]. Both promoter and rho-independent terminators were also experimentally identified in the five homologs of previously identified pneumococcal sRNAs from R6 strain [10]. The results of computational analysis for promoter/terminator showed that most of the sRNAs had a predicted promoter within 25 nt upstream of the TAR start site. In some cases more than one promoter was predicted in the upstream region of sRNA sequence. Rho-independent transcription terminators were predicted for 20 sRNAs within 25 bp downstream of transcription end site. The predicted promoter sequence with transcription start site and terminator sequences for sRNAs are present (Additional file 2). We also evaluated the potential protein coding capacity of sRNAs by translating the sequences in all three open reading frames. Our results indicate that two sRNAs (SN48 and SN50) encoding regions have the potential to code smaller proteins. Further analysis of the DNA sequence in these regions using "FGENESB" gene prediction tool http://www.softberry.com identified the presence of smaller ORF (open reading frame). We did not find any predicted promoter sequences in the upstream regions of these two sRNAs, suggesting they may constitute part of an operon. Further analysis revealed that SN48 is indeed located in a four gene operon (SP1166 to SP1169). BLAST based sequence searches against non-redundant protein database at NCBI did not identify any matches for these two sRNAs in other genomes suggesting that these potential novel genes are currently unique to *S. pneumoniae *TIGR4. While SN48 and SN50 could encode proteins, in absence of experimental validation of ORF, it is not possible to rule out their functional involvement as a sRNA. Therefore we included SN48 and SN50 in our sRNA list (Table 1).

### Comparative genomics of sRNA sequences

The average GC content of sRNAs (35% ± 5%) was slightly less than the average GC content of the TIGR4 genome (39.7%). BLAST analysis of sRNA sequences against the non-redundant nucleotide database at NCBI revealed that all sRNA sequences were highly conserved (coverage ~ 100%, identity > 97%) within other pneumococcal strains (including CGSP14, G54, Hungary19A-6, R6, and D39; Table 1). But only 25 is found to be conserved in closely related species of *Streptococcus *(for example *S. mitis, S. gordonii*, and *S. sanguinis *SK36) [30]. However, these sRNAs were not conserved in other species of *Streptococcus *like *S*. *pyogenes*, *S*. *mutans*, or *S. bovis*. This lack of sRNA sequence conservation at the genus level indicates that these sRNAs might have been acquired during pneumococcal evolution. Six sRNA sequences were found to be conserved in other species outside *Streptococcaceae *(for example *Lactobacillus, Clostridium*, and *Bacillus*) and are known to be involved in various regulatory functions.

### Computational functional prediction of sRNAs

sRNAs can be functionally characterized as either cis- or trans- regulators based on the location of their target genes. The Rfam database [31] is a collection of non-coding RNA families represented by multiple sequence alignments and profile stochastic context-free grammars. We searched all TIGR4 sRNA sequences against the Rfam database to determine their putative functions. We found that some of the pneumococcal sRNAs we identified were homologs to well characterized sRNAs in other genomes. The identified functional categories include FMN riboswitches, TPP riboswitch, PyrR family, Tbox leader elements, r-protein leader autoregulatory structure, putative endoribonuclease (RNaseP_bact_b), tmRNA, and 6S (Table 1; description of individual categories is available at Rfam). With Rfam database searches we could assign putative functions to 14 sRNAs, 11 of which were predicted to be cis-regulators. Three of the cis-sRNAs were predicted riboswitches that could directly bind a small target molecule. For 36 sRNAs we could not predict function using computational methods. These sRNAs likely represent a novel set of non-coding sRNAs in pneumococci.

### Motif and structural analysis of sRNA sequences

**Figure 3:**
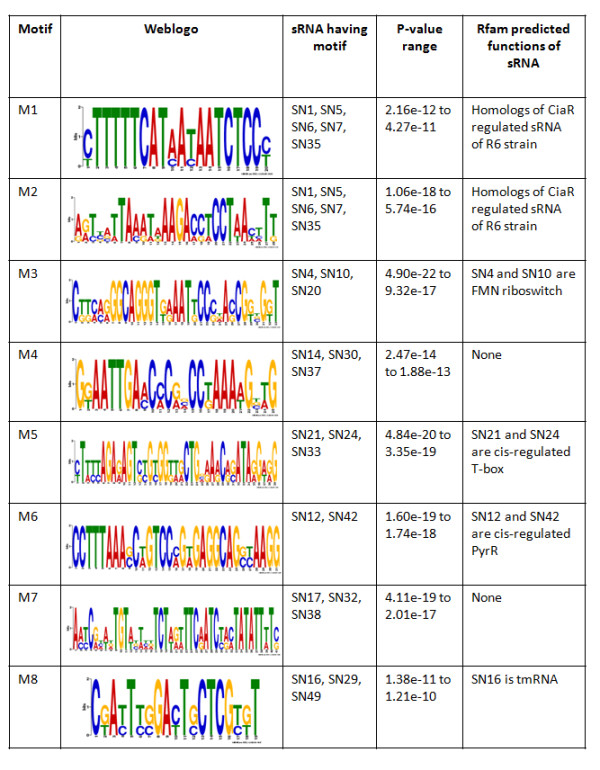
**Sequence motifs identified in sRNAs by MEME**. Overrepresented sequence motifs among non-aligned sRNA sequences were identified by MEME. Rfam annotation for sRNAs are shown where available.

To identify sequence characteristics among the pneumococcal sRNAs, we searched for the presence of overrepresented sequence motifs using MEME SUITE [32]. A sequence motif is a nucleotide sequence pattern that is widespread and has, or is predicted to have, structural or biological significance. All sRNA sequences were used for motif prediction, and the top 8 motifs (present in total 22 sRNAs) were selected based on high score, length (> 15 nucleotides), and p-value (< 1e^-10^). Our results indicate that sRNAs predicted to have similar functions share common motif sequences (Figure 3). All members of motif group M1 and M2 were functionally similar, the five sRNAs which were homologs of CiaRH regulated sRNA in *S. pneumoniae *R6 strain. Similarly, members of motif group M3, M5 and M6 share similar functions.

We also investigated secondary structure of motif sequences based on MFOLD predicted sRNA structures [33]. Our results showed that in most motif groups, the sRNA sequences had similar motif structures (Additional file 4). Motif M1 always forms a partial stem loop-like structure in all five sRNAs (SN1, SN5, SN6, SN7, and SN35), while motif M2 forms a large unpaired segment. Motif M2 in SN7 and SN35 assumes a partial stem loop structure while a large portion of the sequence still remains unpaired. Motifs M3, M4, M5, and M7 form stem loop structures in corresponding sRNA sequences. Motifs M6 and M8 includes two stem loop structures along with the unpaired region between them. The 28 sRNAs that had no detectable sequence motifs could represent a set of diverse sequences having different mode of action.

Searching these motifs in motif database using TOMTOM [34] results in identification of motif M6 associated with pyrR (transcriptional attenuator and uracil phosphoribosyltransferase activity) regulated function, similar to sRNAs (SN12 and SN42) predicted function. Motif M6 was identified to be a part of antiteminator binding region in regulatory protein, PyrR, where it regulates the transcription of pyr operon by attenuation mechanism [35-37]. We also analyzed two motifs M3, M5 that were present in the sRNAs whose functions are well described in literature. Motif M3 was found to be a part of aptamer structure (the region binding to small molecules) of FMN riboswitches [38,39]. Motif M5 was found to be present in the conserved part of the specifier loop of T-box regulated genes [40,41]. T-box antitermination is considered as one of the main mechanisms to regulate gene expression in amino acid metabolism in gram-positive bacteria. The other described motifs could represent important novel structural or functional regions to be investigated.

### Gene expression profile and identification of operon structures

**Figure 4:**
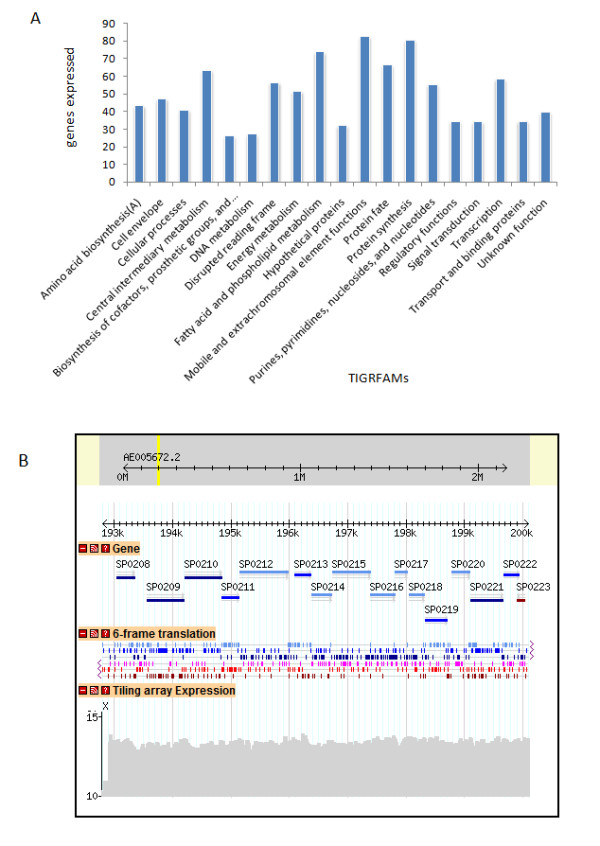
**A *S. pneumonia*e TIGR4 genes expressed in different TIGR protein families (TIGRFAMs)**. The gene expression is shown as a percentage of the total number of genes present in TIGR4 genome in a particular TIGRFAM category. **B. Genome browser visualization of S10, a 15 gene operon (SP0208 - SP0222)**. Track two shows the DNA sequence translation in six frames and track one shows the genes. The color of the expressed genes is in accordance with the six frame translation. S10 operonic genes SP0208 - SP0222 are present in the forward strand. The "tiling array expression" track clearly demonstrates that all genes predicted in S10 operon are expressed at similar level and this expression is higher than the intensity threshold for expression (11.0).

Our results indicate that ~50% of *S. pneumoniae *TIGR4 genes were expressed during mid-log growth phase. We characterized the set of expressed genes, which represent basal transcriptional activity under our growth condition, using TIGRFAMs (Figure 4A). The expressed genes are involved in fundamental biological processes such as transcription, protein synthesis, protein fate, and cell division (Additional file 5). Processes such as fatty acid and phospholipid metabolism were also represented in the expression profile. We found that approximately 40% of the expressed genes were involved in processes mediating DNA metabolism, regulatory functions, and signal transduction. Almost all genes with mobile and extra-chromosomal functions were expressed. Genes encoding surface proteins, proteins involved in acquiring nutrients, and transporters were also expressed [24]. Interestingly, one third of the annotated hypothetical genes (97) and around half of the genes annotated as disrupted reading frames (52 out of 92) were expressed.

In bacteria genes involved in carrying out similar function are often organized into operon structures. Identifying operon structures is critical for understanding coordinated regulation of bacterial transcriptome. Identifying transcriptional units can also help in assigning function to hypothetical genes when present in an operon of known function [42]. Tiling arrays efficiently identify co-expressed genes and transcription units at a genomic scale. We identified co-expression for 520 pairs of TIGR4 genes (Additional file 6) that were transcribed together and constituted minimal operons. By joining consecutive overlapping pairs of co-expressed genes, we identified 202 distinct transcription units/operons (size varied between two to fifteen genes; Additional file 7).

The operons identified in this study were compared to previously described pneumococcal operons (Table 2). The *vic*, *man*, *atp*, and *marMP *operons identified by tiling arrays concur with previously described operon structures [43-46]. In *S. pneumoniae *R6, *marMP *operon is considered to have three genes (SP2108-SP2110 in *S. pneumoniae *TIGR4). In contrast, our results identified only two genes as transcription unit (SP2109-2110). Our data clearly shows that the expression of SP2108 is higher than SP2109 - SP2110 (Additional file 8), suggests that SP2108 is either expressed as an independent transcription unit or there exists a possibility of overlapping transcripts among these three genes. We did not identify *murMN*, *phg*, and *comCDE *operon expression, suggesting that these genes may not be required for mid-log growth phase. Lack of expression of competence related genes is expected as THB medium used for propagating *S. pneumoniae *does not support competence.

**Table 2:** Comparison of *S. pneumoniae *TIGR4 operons identified by tiling arrays with Streptococcus operons described in literature.

Operon name	Experimental prediction	Tiling array predictions	Literature reference (PUBMED ID)
murMN	SP0615-SP0616	-	10759563

vic	SP1225-SP1226-SP1227	SP1225-SP1226-SP1227	12379689

MiaR reg MarMP (3 operon)	SP2106-SP2107	SP2106-SP2107	
		
	SP2108-SP2109-SP2110	SP2108	11278784
		
	SP2111-SP2112	SP2109-SP2110	
		
		SP2111-SP2112	

phg	SP1043-SP1044-SP1045	-	15271918

TrmD	SP0776-SP0777-SP0778-SP0779-SP0780	SP0778-SP0779-SP0780	15060037

ComCDE	SP2235-SP2236-SP2237	-	9352904

luxS & Dcw3	SP0340	SP0340	16436421
		
	SP0334-SP0335-SP0336-SP0337	SP0336-SP0337	

man	SP0282-SP0283-SP0284	SP0282-SP0283-SP0284	12486041

atp	SP1507-SP1508-SP1509-SP1510-SP1511-SP1512-SP1513-SP1514	SP1507-SP1508-SP1509-SP1510-SP1511-SP1512-SP1513-SP1514	15576803

Comparing our experimentally identified co-expressed genes with computationally predicted operons using "DOOR" [47] showed that there was an approximately 63% overlap between both datasets (291 gene pairs, excluding rRNA and tRNA; Additional file 9). Thus, our dataset experimentally validates 291 DOOR gene-pair predictions. Tiling array expression analysis also identified 229 additional co-expressed gene-pairs that were not predicted by DOOR, which may help in refining the boundaries of identified transcriptional units with greater accuracy. For example, DOOR predicted the S10 operon (coding for ribosomal proteins) in TIGR4 as a 14 gene operon (SP0209-SP0222). However, tiling analysis indicated that the S10 operon has 15 genes (SP0208-SP0222) and included SP0208 (Figure 4B). Interestingly, we found that *Bacillus subtilis *S10 operon structure is similar to our experimentally derived pneumococcal S10 operon structure (fifteen genes, including a SP0208 homolog) [48]. One possible reason for the exclusion of SP0208 by DOOR could be the relatively large 217 bp intergenic region between SP0208 and SP0209. In another example, tiling expression identified *rplK-rplA *(SP0630-SP0631) genes as part of single transcriptional unit, but DOOR failed to identify this unit possibly due to the presence of a large 207 bp intergenic region *between rplK *and *rplA*.

Proteins encoded by genes in the same operon often have related function or are in the same biological pathway. Therefore, putative function may be assigned to hypothetical genes when located in an operon of known function [42]. In our operon dataset, approximately 20% (147) of the genes encode hypothetical proteins. In operon 8, a three gene operon (SP0077 - SP0079), two genes encode Trk family of potassium uptake proteins, and one gene (SP0077) encodes a hypothetical protein. Therefore, it is possible that SP0077 may be a member of the Trk transporter protein family. In another three gene operon (SP0904-SP0906), all genes encode hypothetical proteins; it is possible these proteins have similar as yet un-assigned functions.

### Experimental validation of sRNAs

Expression of 14 sRNAs identified by genomic tiling expression analysis was analyzed by qRT-PCR. The sRNAs selected for validation included 5 sRNAs identified in *S. pneumoniae *R6 strain and 9 novel TIGR4 sRNAs identified in the current study. Statistical t-tests were performed for each sRNA between the C_t _value for the (reverse strand) vs the C_t _value of the background (no primer) to determine if there was significantly higher expression than background. Another t-test was conducted for each sRNA between the C_t _value for the reverse strand vs the C_t _value for the forward strand to determine if there was significant expression from the sense strand. At *p *≤ 0.05, for 13 sRNAs we found significantly higher expression (lower C_t _value) for the coding strand specific qRT-PCR compared to the non-coding strand and background (no primer) (Additional file 10). The *p*-value of sRNA SN24 was not significant at *p *< 0.05. Three of the validated sRNAs (SN4, SN12 and SN16) had available annotations (Table 1). Although validated, no functional information was predicted for sRNAs SN2, SN11, SN22 and SN27. All five sRNAs whose homologs were present in *S. pneumoniae *R6 strain were also positively validated. Overall, qRT-PCR validations were successful for thirteen out of fourteen sRNAs.

## Discussion

Tiling array analysis is widely used in eukaryotes to study transcriptional complexity and identifying non-coding transcripts [49-52]. Recent studies in *Mycobacterium leprae *and *E.coli *described whole genome tiling array approach for sRNA identification [20]. Parallel sequencing technology was used for sRNA identification in *Salmonella *[23] and *Vibrio cholerae *[22]. Individual experimental studies [10,15] altogether identified 14 sRNAs in two different strains of *S. pneumoniae *(D39 and R6 strain). To our knowledge, this is the first study to report the use of whole genome tiling arrays for experimental identification of sRNAs at a global scale in *S. pneumoniae*. The tiling array analysis method described here is a combination of the methods described by others [49,52], but tailored for prokaryotic genomes. Hfq protein plays a central role in sRNA function in *E. coli*, facilitating the pairing of sRNA with its mRNA target [53]. One experimental approach for sRNA identification in bacteria could be the co-immunoprecipitation of sRNA using Hfq antibodies [16]. However, *S. pneumoniae *TIGR4 genome does not code for Hfq protein which precludes applying this method to TIGR4 genome. Therefore, tiling array approach described in this study is a pragmatic experimental approach for identifying sRNAs. Identifying the sRNA repertoire of TIGR4 is the first step towards understanding the sRNA regulatory network of this human pathogen.

The transcriptome map generated in this study identified expression in two thirds of TIGR4 genome. Tiling array analyses of *E. coli *and yeast reported expression of 87% and 90% of the genome respectively [50,54]. Compared to these studies, TIGR4 genome expression in this study was relatively in lower proportion (68%). Possible reasons for this lower expression could be the growth conditions and/or the stringent intensity cutoff used for identification of expressed regions. We choose a stringent intensity cutoff (11.0) to maintain a low false positive rate (1.63%) for identifying sRNAs, which are usually short in length (50-200 bp).

As a result, we report for the first time genome-wide identification of 50 novel sRNAs in pneumococcus using tiling arrays. Additional features, such as presence of a promoter and rho-independent terminator, were computationally predicted for identified sRNAs. Almost half of the identified sRNAs showed the presence of a rho-independent terminator. As speculated by others [29,55], our analysis indicates that identification of rho-independent terminator sequence is the strongest determinant for the identification of sRNA. Furthermore, the identification of rho-independent terminator downstream from sRNA sequences helped us in differentiating the sRNAs from the 5' untranslated extensions of genes. However, it is possible that some sRNAs may be associated with a rho-dependent terminator and thus would not be identified in our search.

Comparative genomics of sRNA sequences revealed that only six sRNA sequences involved in various regulatory activities were conserved beyond *Streptococcaceae *(example *Lactobacillus, Clostridium, Bacillus *(Table 1). The evolutionary tree of Streptococcus family [30] indicates that *S. mitis, S. gordonii, S. sanguinis SK36 *are phylogenetically closer to *S. pneumoniae *than other species (like *S. pyrogens, S. mutans or S. bovis) *which explains the conservation of 25 sRNAs in *S. mitis, S. gordonii*, and *S. sanguinis *SK36), but not present in other species like *S*. *pyogenes*, *S*. *mutans*, or *S. bovis*.. It also indicates that sRNA prediction algorithms that rely on comparative genomics need to first account for the observed low sequence conservation of sRNAs among different species [13]. Our results suggest that computational methods which rely on comparative genomics to find sRNAs need to focus on carefully selected closely related species. The 50 sRNAs identified in this study along with their comparative genomics could serve as a training dataset for further computational sRNA predictions in pneumococcus, particularly for the identification of sRNAs which are not expressed under our experimental conditions. At last, we speculate that computational prediction of *Streptococcus *sRNAs using comparative genomics with *S. mitis*, *S*. *gordonii*, and *S*. *sanguinis *SK36 will identify new as yet undescribed sRNAs.

Exploring the sequence characteristics of sRNAs described in this study showed that sRNAs predicted to have similar biological function share common sequence motif. We identified 8 sequence motifs, of which five were identified in TIGR4 for the first time. Members of the motif group without predicted function could have similar structural or functional properties. For example SN20 had motif M3 and might function as a FMN switch similar to SN4 and SN10, which also contain this motif. Likewise, sRNAs present in motif group M4 could be predicted to have similar yet undefined function. Structural analysis of motif (Additional file 4) suggests that they mainly form two kinds of structure in sRNAs; firstly, the whole motif forms a stem loop structure (like motif M5) and secondly, the motif is present as two stem loop structures including the unpaired region between them (like motif M6). Furthermore, motifs present in sRNAs with similar function also formed a conserved secondary structure (for example, motifs M1, M2, and M5). We speculate that (SN32 and SN38), (SN16 and SN29), (SN21, SN24 and SN33), (SN14 and SN37) contains similar motif structure and might share similar yet unknown structure/function. This structural conservation of motifs also suggests that motif regions of sRNA could be structurally or functionally important regions and can be used as targets for mutational studies to decipher function.

The accuracy of computational operon prediction in bacteria is 85-91% in terms of specificity and sensitivity for predicting operonic gene pairs (pairs of consecutive genes that are part of the same operon) in *E. coli *and *B. subtilis*, respectively [56]. However, the sensitivity of prediction drops to as low as 50% when predicting transcription units with more than one gene [56]. Two examples were discussed in results where the computational prediction failed to identify a gene pair as a part of an operon due to the presence of a large intergenic region between them. The accuracy of computational operon prediction algorithms also decreases when performing predictions for newly sequenced genomes for which no training dataset is available. Based on tiling array analysis, we generated 520 gene pairs that were co-expressed and identified 202 transcription units in *S. pneumoniae *TIGR4. Our results clearly demonstrate the effective use of tiling arrays for operon identification at a whole genome scale. An obvious limitation to the tiling array approach is the inability to identify operons whose genes are not expressed in the experimental growth condition. Nevertheless, our results demonstrate that combining operons identified by tiling with computational prediction greatly improves operon identification in genomes, as speculated by other researchers [57]. The operons identified in this study, though not comprehensive, still represent a validated dataset of approximately 202 operons.

Around 8% of the *S. pneumoniae *TIGR4 genome is repetitive in nature. It includes sequences (> 50 bp) that are present at multiple locations in the genome, such as mobile genetic elements, small dispersed repeats like RUP and BOX elements, and other repetitive regions. Although these regions were excluded for identifying sRNA, we detected a high level of transcription in these repetitive regions from both sense and antisense strands. Because it is not possible to identify the actual origin of transcription with tiling arrays, future experiments designed to analyze the transcriptional activity in these repeat regions are warranted. In view of recent findings where sRNAs are involved in repressing expression of toxic proteins [58] and are present in multiple copies, we speculate that that these repetitive regions may be involved in various regulatory activities within the cell.

In conclusion, our combinatorial approach of experimental identification of sRNAs on a genome scale using tiling arrays in conjunction with computational analyses of sRNAs in *S. pneumoniae *TIGR4 has resulted in the description of 50 sRNAs in this clinically relevant strain. Our result forms the initial framework for understanding sRNA-based regulation of *S. pneumoniae *gene expression.

## Conclusions

Here we have demonstrated the utility of tiling arrays to study whole genome transcription in prokaryotes. The analysis of high-resolution transcription map of the *S. pneumoniae *clinical isolate TIGR4 results in identification of 50 novel sRNAs. Bioinformatics sequence based searches helped to predict function of 14 sRNAs. Comparative genomics shows that half of the identified sRNA sequences are unique to *S. pneumoniae *genome. We identified eight overrepresented sequence motifs among sRNA sequences that correspond to different functional categories. We identified 202 operon structures in the genome, further validated by available experimental identifications. Overall, this work elucidated pneumococcal operon structures and identified previously undiscovered sRNAs, which will enhance our understanding of the complexity and extent of the pneumococcal 'expressed' genome. Also, this work opens up new avenues for understanding the complex RNA regulatory network governing *S. pneumoniae *physiology and virulence.

## Methods

### Isolation of total RNA from *S*. *pneumoniae *TIGR4

*S. pneumoniae *strain TIGR4 [24] was grown in Todd-Hewitt broth supplemented with 0.5% yeast extract (THY). Cells were harvested during mid-log phase (OD_600 _nm, 0.4-0.5) of growth by centrifugation from two biological replicates. The harvested pellets were washed twice in sterile phosphate-buffered saline (PBS; pH 7.4) and stored at -80°C. RNA was purified from frozen bacterial pellets using Qiagen RNeasy kit http://www.qiagen.com/ following the manufacturer's protocol. Isolated RNA was treated with DNase, and the purity was checked by performing a one-step RT-PCR using primers specific for 16 S rRNA in the presence or absence of reverse transcriptase. RT-PCR performed in the presence reverse transcriptase in the reactions resulted in the amplification of the desired PCR product. In contrast, no PCR product was generated when reverse transcriptase was excluded from the reaction mix, confirming that the isolated RNA did not have genomic DNA. RNA concentration and quality were determined by using Agilent Bioanalyzer (Agilent, Foster City, CA). Purified RNA was stored in nuclease free water at -80°C. One microgram of total RNA was used by Nimblegen systems (Roche NimbleGen, Inc. Madison, WI) for labeling and hybridization.

### High density genome tiling and hybridization

High density oligonucleotide microrrays from Nimblegen Systems that incorporate "Maskless Array Synthesis" [59] technology for designing probes were used to study the expression of TIGR4 genome. The tiling array was designed based on the TIGR4 genome sequence (obtained from Genbank, accession number NC_003028). Probes of 50 nucleotide length were designed in an overlapping fashion at 12 bp intervals for both strands across the entire genome, resulting in a total of 359,366 probes. Twenty thousand random probes were included for measuring non-specific hybridizations. Labeling of cDNA with Cy3, hybridization, and scanning were conducted by Nimblegen Systems (detailed protocol available at http://www.nimblegen.com/products/lit/lit.html) and Nimblegen provided resulting raw fluorescence intensity values.

### Normalization and data analysis

Spatial effects (uneven washing or scanning) were removed from the fluorescence intensity data using a global distance-weighted smoothing algorithm for correction available in the NimbleGen Microarray Data Processing Pipeline (NMPP) [60]. NMPP output was log transformed for further analysis. Quantile normalization was performed using the Affy package available in R language http://pbil.univ-lyon1.fr/library/affy/html/normalize.quantiles.html to remove systematic errors (biases) from the replicate slides and to generate identical intensity distribution for both chips [61]. The correlation coefficient between the intensities of the two chips was r^2 ^≥ 0.90.

Although a number of methods are described in the literature for tiling array data analysis [62-65], most were not readily applicable to our dataset because of our single color array design. Furthermore, the existing methods are not tailored for prokaryotic genomes. Therefore, for processing our TIGR4 tiling array data, we modified Kampa *et al. *method [52] as described below:

1. Instead of using PM (positive-match) - MM (mis-match) intensities, we used PM probe intensities only.

2. Pseudomedian filter (which takes adjacent probe intensities into account) was used to adjust for sequence based variation at the probe level and provide an initial smoothing of the raw probe intensity values [26]. Pseudomedian (Hodges-Lehman estimator) for each probe was calculated with a sliding window size of 11 probes (170 bp).

3. To identify the transcribed regions of the genome, we considered a probe to be expressed when its pseudomedian intensity was found to be higher than a threshold value. The threshold value was determined on the basis of distribution of positive and negative control probe intensities, pseudomedian filter setting, accuracy of transcript boundary detection, and the associated false positive rate.

4. To identify TARs (transcriptionally active regions), consecutive expressed (transcribed) probes were joined together using maxgap-minrun method [52]. The maxgap parameter allows certain number of probes (one or two probes) to be below the cutoff while still being incorporated into the TAR, whereas the minrun parameter requires at least a certain length of the TAR to be considered further. To account for the densely packed prokaryotic genomes (shorter intergenic regions), the maxgap feature was not applied in the intergenic regions. We used a minrun value of 74 (at least 3 consecutive probes) for sRNA detection.

5. The pseudomedian filter can result in slightly erroneous identification of transcript boundaries (start and end). Therefore, we implemented a new step that re-modified transcript boundaries using normalized average raw intensity values. Re-modification was conducted by either elongating or shortening transcript ends until the average raw intensity values of the probe (not pseudomedian value) was greater than or equal to the threshold cut off. Overlapping transcripts were then joined together for TAR generation.

All of the above analytical steps were performed using in-house PERL scripts. Steps one, four, and five were modifications of the Kampa method and are specific to our analysis. The tiling array data from this study have been submitted to Gene Expression Omnibus under accession no. GSE12636.

### Analysis of annotated regions of TIGR4 genome

#### Gene expression

Identified TARs were mapped to the current annotation of *S. pneumoniae *TIGR4 genome [24]. We found that each gene was represented by a mixed set of expressed and non expressed probes. Genes that had a significantly higher proportion of expressed probes in a binomial test [50] were considered to be expressed (*p *< .001, which results in at least 70% gene length coverage by TAR). This set of expressed genes represented the basal transcription of TIGR4. Functional analysis of the expressed genes was conducted based on "TIGRFAMs" http://www.tigr.org/TIGRFAMs/index.shtml.

#### Operons

Because tiling arrays measure expression in the intergenic regions of annotated genomes, they can be used to identify and predict operon structures in bacteria. Two or more consecutive genes were considered to be part of an operon, if they fulfilled the following criteria: (a) they are expressed, (b) they are transcribed in same direction, and (c) the intergenic region between the genes was identified as a single expressed transcript that overlapped the genes in both directions. Overlapping pairs of genes are joined together to identify large operon structures.

### sRNAs identification, genomic and structural analysis

To identify small RNAs, TARs were mapped to intergenic regions of the *S. pneumoniae *chromosome. Intergenic regions within operons, small 5' and 3' untranslated extensions (UTR) of mRNAs, and non-unique regions (mobile genetic elements and repetitive regions) of the genome were excluded. Only sRNAs that were identified at a minimum length of 74 bp (3 consecutive probes) were considered. Additional features for sRNAs such as promoters and transcription terminators were predicted computationally to add confidence in their identification. Bacterial promoter prediction was done using the "Neural Network Promoter Prediction" program http://www.fruitfly.org/seq_tools/promoter.html[66]. Putative sRNA sequences including 50 base pair upstream region were utilized for promoter prediction. Rho-independent transcription terminators were identified using program TransTermHP [67]. The putative sRNA sequence with 50 base pair downstream region is included for terminator prediction. UTR regions of length less than 100 bp are discarded. Variation in transcriptional intensity, presence of promoter and presence of rho-independent terminators are used as evidences to identify structural regulatory elements located inside the leader sequences. A circular *S. pneumoniae *TIGR4 genome map along with genes and sRNAs was generated using DNAPlotter [68]. All sRNA sequences were searched against Rfam database [31] for functional annotation. BLASTN searches were performed against non redundant nucleotide database at NCBI to determine sRNA sequence conservation among other genomes. MEME [69] was used for the identification of motifs in non-aligned sRNA sequences, where a motif is a sequence pattern that occurs repeatedly in a group of nucleotide sequences. Selected motifs were searched for their presence against the preexisting motif database using TOMTOM [34]. Sequence logos for predicted motifs were generated by WebLogo [70]. sRNA secondary structures were predicted using MFOLD [33]. The sRNAs, along with additional features, were mapped onto the TIGR4 genome in Genome Browser "GBrowse" [71]http://gbrowse.lsbi.mafes.msstate.edu/cgi-bin/gbrowse/TIGR4/ for visualization, analysis, and web based accessibility.

### qReal-time PCR

Expressions of 13 sRNAs were validated by complementary quantitative Reverse Transcription - Polymerase Chain Reaction (qRT-PCR). PCR primers were designed (Additional file 11) using Primer3 [72] with at least one GC clamp on the 3" end. The same RNA used for tiling array labeling and hybridization was used as the template for qRT-PCR. All reverse transcription (RT) and subsequent PCR reactions were done in parallel and in triplicate. For each sRNA, three different RT reactions were set up. To measure possible expression of each complementary DNA strand, two strand-specific RT reactions were done; each reaction used only one strand-specific primer (forward or reverse). The third RT reaction was conducted in the absence of primers (to account for primer independent cDNA synthesis). After the RT step, both primers were added to all three reactions to complete the PCR step. RT-PCR was performed with 10 ng *S. pneumoniae *RNA using the Platinum^® ^SYBR^® ^Green One-Step qRT-PCR Kit (Invitrogen Corporation, Carlsbad, CA) as described [73]. Briefly, strand-specific RT reaction was conducted at 50°C for 10 min, 95°C for 5 min, and 0°C for 5 min. At this stage, the PCR primers were added to the reaction, and amplification and detection of specific PCR products was accomplished using the iCycler iQ Real-Time PCR Detection System (Bio-Rad Laboratories, Inc., Hercules, CA) with the following cycle profile: 95°C for 5 min, followed by 45 identical cycles at 95°C for 15 s and 60°C for 1 min. Melt curve analysis used 95°C for 1 min and 55°C for 1 min, followed by 80 cycles of 55°C for 10 s. The C_t _(threshold cycle) values from all three RT-PCR reactions in triplicate were analyzed to detect sRNAs expression (Additional file 10).

## Authors' contributions

RK designed the analysis workflow with BN, wrote all the scripts required for the analysis, carried out data analysis and wrote the initial draft of this manuscript. PS prepared the RNA samples for tiling array analysis and helped in data interpretation and initial draft preparation. ES, SB and ML and BN conceived and designed this collaborative study, helped with data analysis and interpretation. ES, SB and ML and BN helped draft the final version of the manuscript. All authors read and approved the final manuscript.

## Supplementary Material

Additional file 1 **Determination of intensity threshold for probe expression**. Distribution of the intensities for positive and negative control probes was used to determine the threshold cutoff for probe level expression. Click here for file

Additional file 2 **Genomic features of identified sRNAs**. *S. pneumoniae *TIGR4 sRNAs and their DNA sequences are shown with the transcription start sites (TSS, bold) predicted by "Neural Network Promoter Prediction". For sRNAs that were also identified in strain R6 (SN1, SN5, SN6, SN7 and SN35) the experimental (*) TSS are shown. Click here for file

Additional file 3 **Comparison of sRNAs from different studies**. Comparison of sRNAs identified in this study with previously described sRNAs (using computational and experimental approaches) in *S. pneumoniae*. Click here for file

Additional file 4 **sRNA secondary structure prediction**. Predicted secondary structure of sRNAs using MFOLD. Motif regions are colored. Click here for file

Additional file 5 **Gene expression profile**. *S. pneumoniae *TIGR4 genes identified as expressed in the present study, their associated TIGRFAM roles, sub roles and functions (where available). Click here for file

Additional file 6 **List of co-expressed genes**. Pairs of co-expressed genes in *S. pneumoniae *TIGR4 identified by genomic tiling arrays. Click here for file

Additional file 7 **List of identified transcription units**. Transcription units identified by joining co-expressed gene pairs in *S. pneumoniae *TIGR4. Click here for file

Additional file 8 **GBrowse visualization of a transcription unit**. Genome browser visualization of genes SP2108 - SP2110. The tracks shown include translation in all six frames and tiling array expression. All three genes are present in the forward strand. The "tiling array expression" track clearly shows high level of expression for SP2108 compared to SP2109-SP2110. Click here for file

Additional file 9 **Comparison of co-expressed gene pairs**. Comparison of co-expressed gene pairs identified by tiling arrays with the results of computational operon prediction program "DOOR". Click here for file

Additional file 10 **qRT-PCR validations of sRNAs**. qRT-PCR validations of *S. pneumoniae *TIGR4 sRNAs carried out in triplicate. Click here for file

Additional file 11 **Primer sequences for qRT-PCR**. DNA sequences of primers used for qRT-PCR for validation for sRNAs in *S. pneumoniae *TIGR4. Click here for file
