# Supplementary material for: Identification of novel non-coding small RNAs from Streptococcus pneumoniae TIGR4 using high-resolution genome tiling arrays
Source: BMC Genomics. 2010 Jun 3;11:350. doi: 10.1186/1471-2164-11-350 (PMC2887815; doi:10.1186/1471-2164-11-350)
Supplement: Additional file 1 — Determination of intensity threshold for probe expression. Distribution of the intensities for positive and negative control probes was used to determine the threshold cutoff for probe level expression. [file 1471-2164-11-350-S1.DOC]

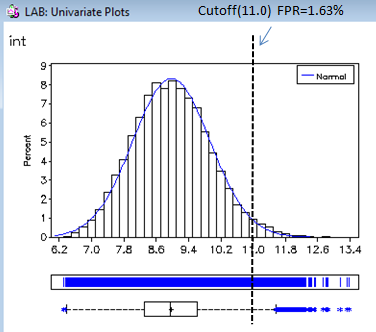

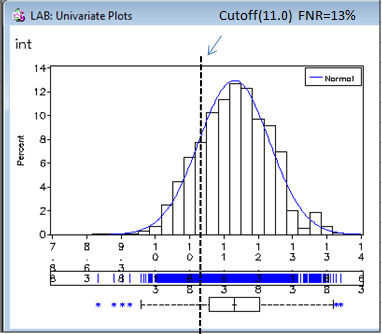


Negative control probes intensity distribution Positive control probes intensity distribution

Supplementary figure. Distribution of the intensities of positive and negative control probes with reference to the intensity threshold cutoff for expression (11.0). The X axis represents intensity values and Y axis represents percentage of probes. The intensity plots were generated in SAS and show normal distribution. At the probe level cutoff value of 11, the false positive rate was 1.63%. The false negative rate associated with this cutoff is around 13%.
