# Supplementary material for: Identification of novel non-coding small RNAs from Streptococcus pneumoniae TIGR4 using high-resolution genome tiling arrays
Source: BMC Genomics. 2010 Jun 3;11:350. doi: 10.1186/1471-2164-11-350 (PMC2887815; doi:10.1186/1471-2164-11-350)
Supplement: Additional file 4 — sRNA secondary structure prediction. Predicted secondary structure of sRNAs using MFOLD. Motif regions are colored. [file 1471-2164-11-350-S4.PPT]

## Slide 1
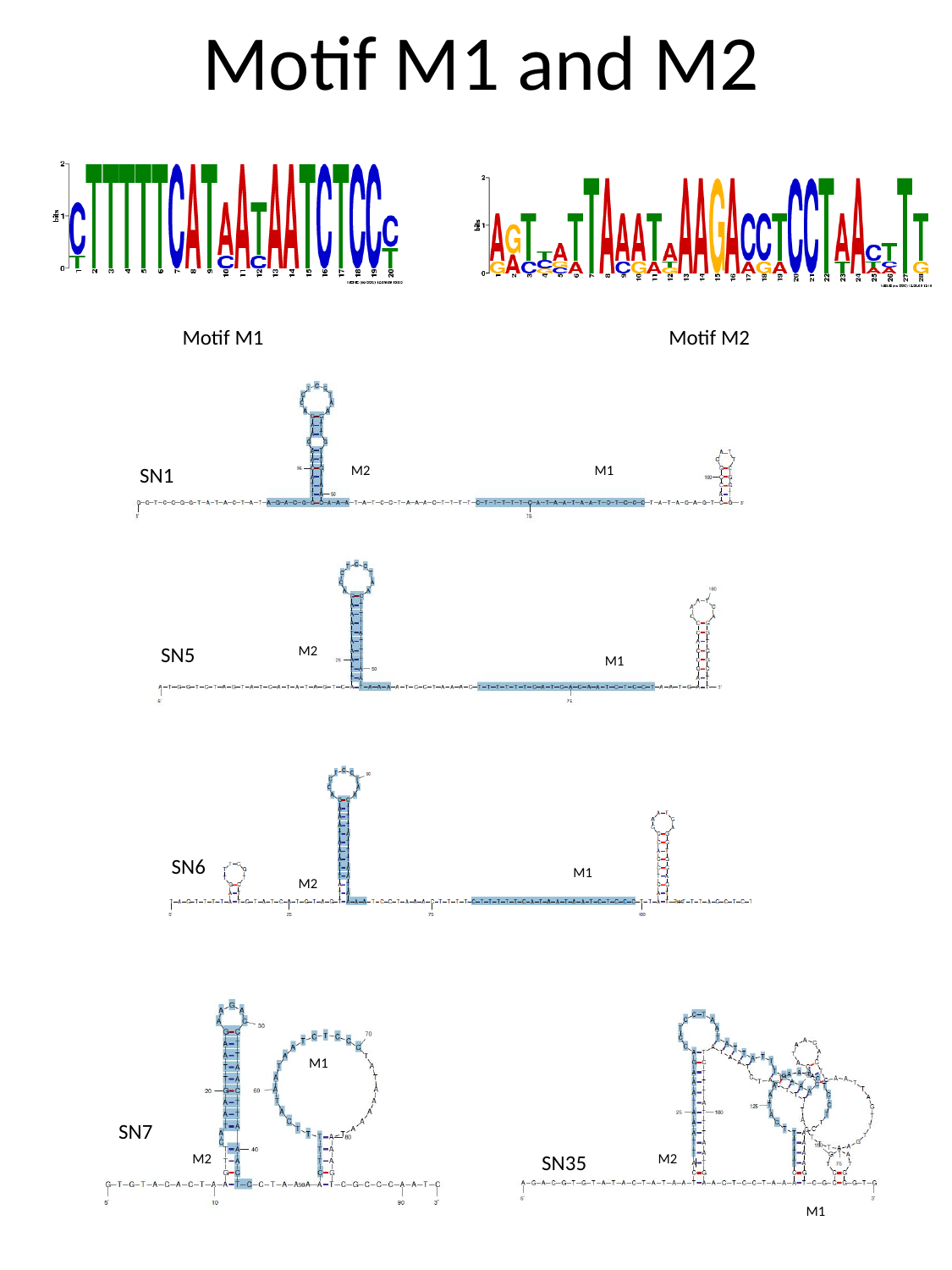

# Motif M1 and M2
Motif M1
Motif M2
SN1
M2
M1
SN5
M2
M1
SN6
M1
M2
M1
SN7
M2
SN35
M2
M1

## Slide 2
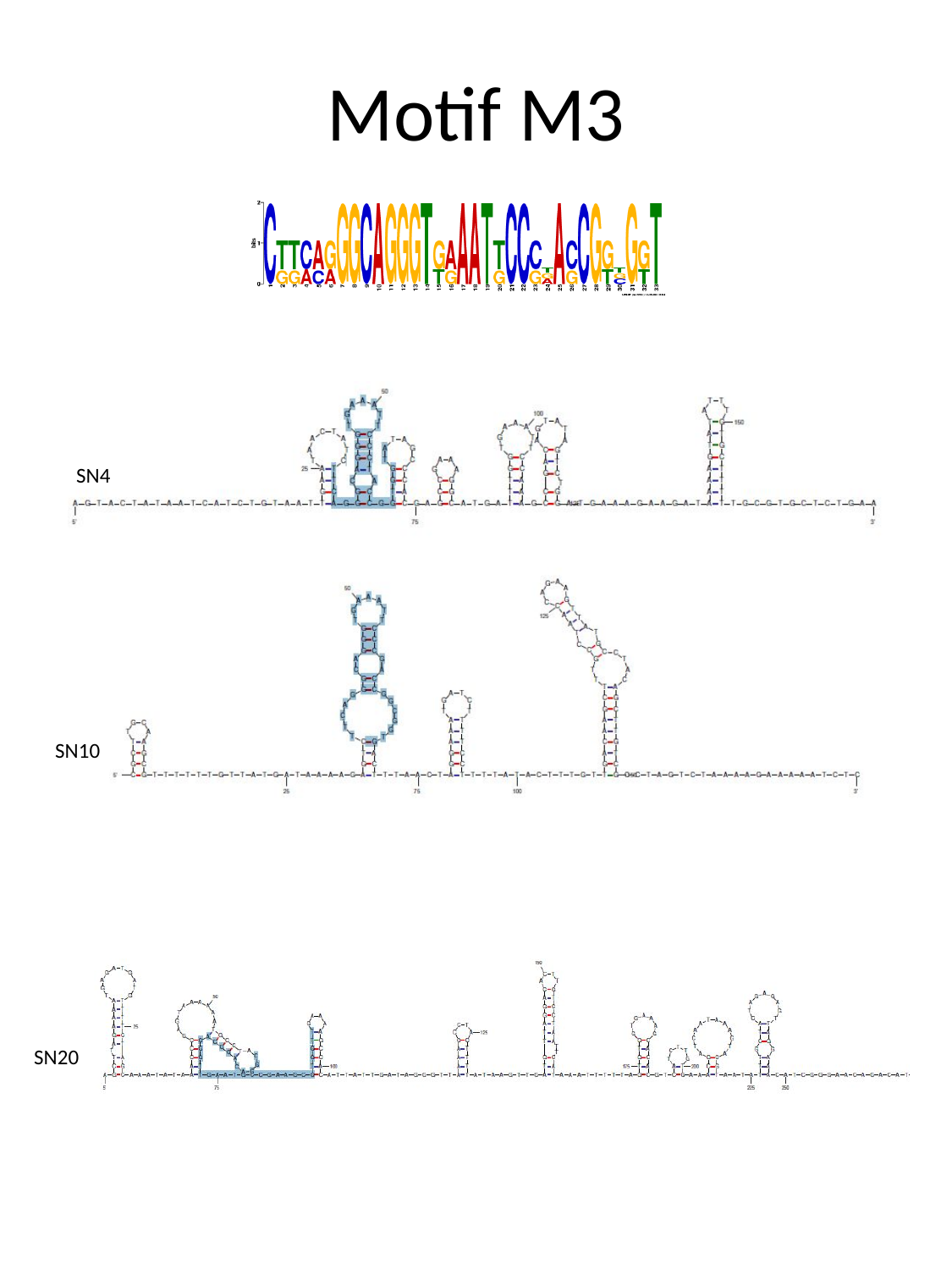

# Motif M3
SN4
SN10
SN20

## Slide 3
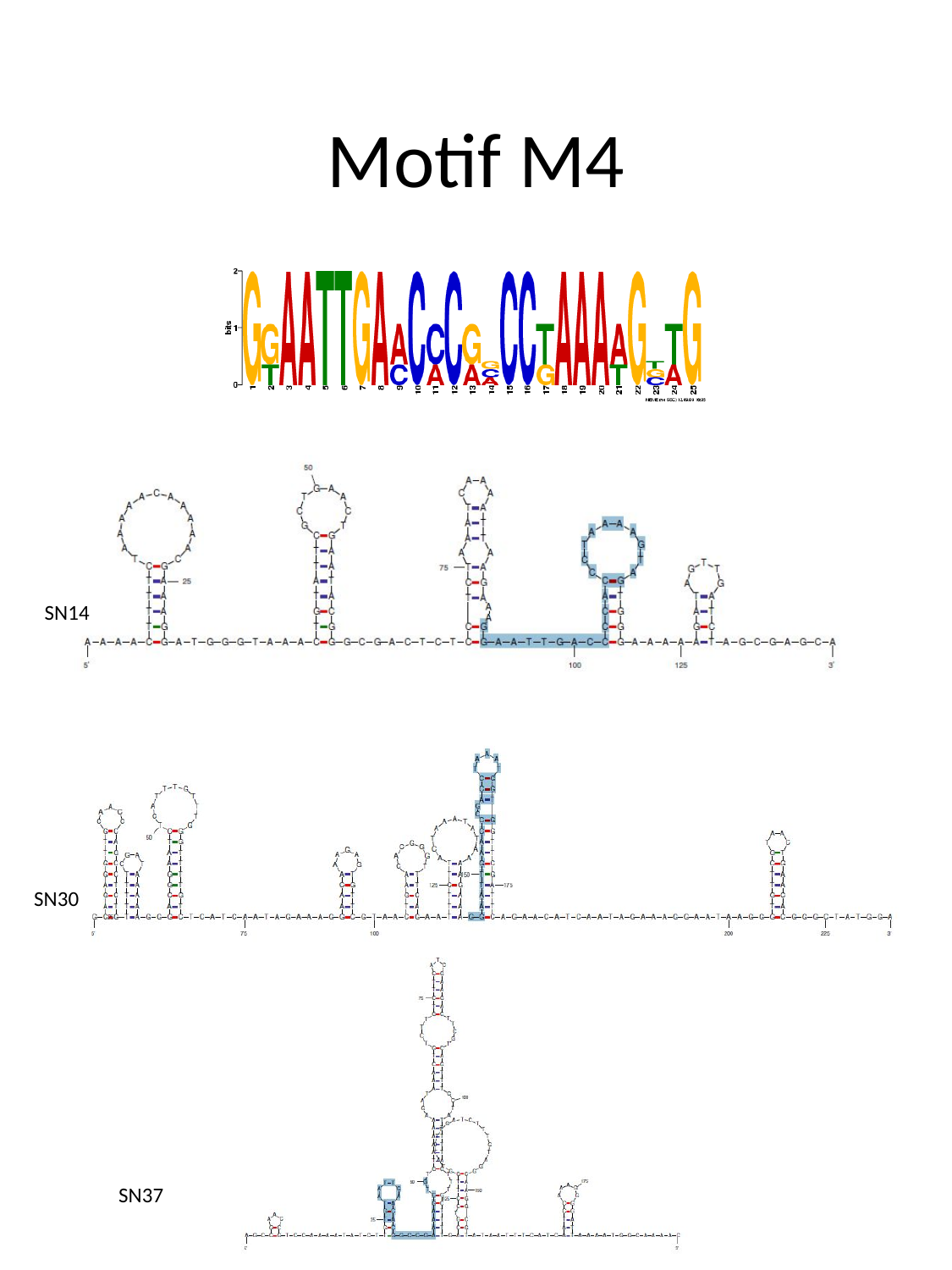

# Motif M4
SN14
SN30
SN37

## Slide 4
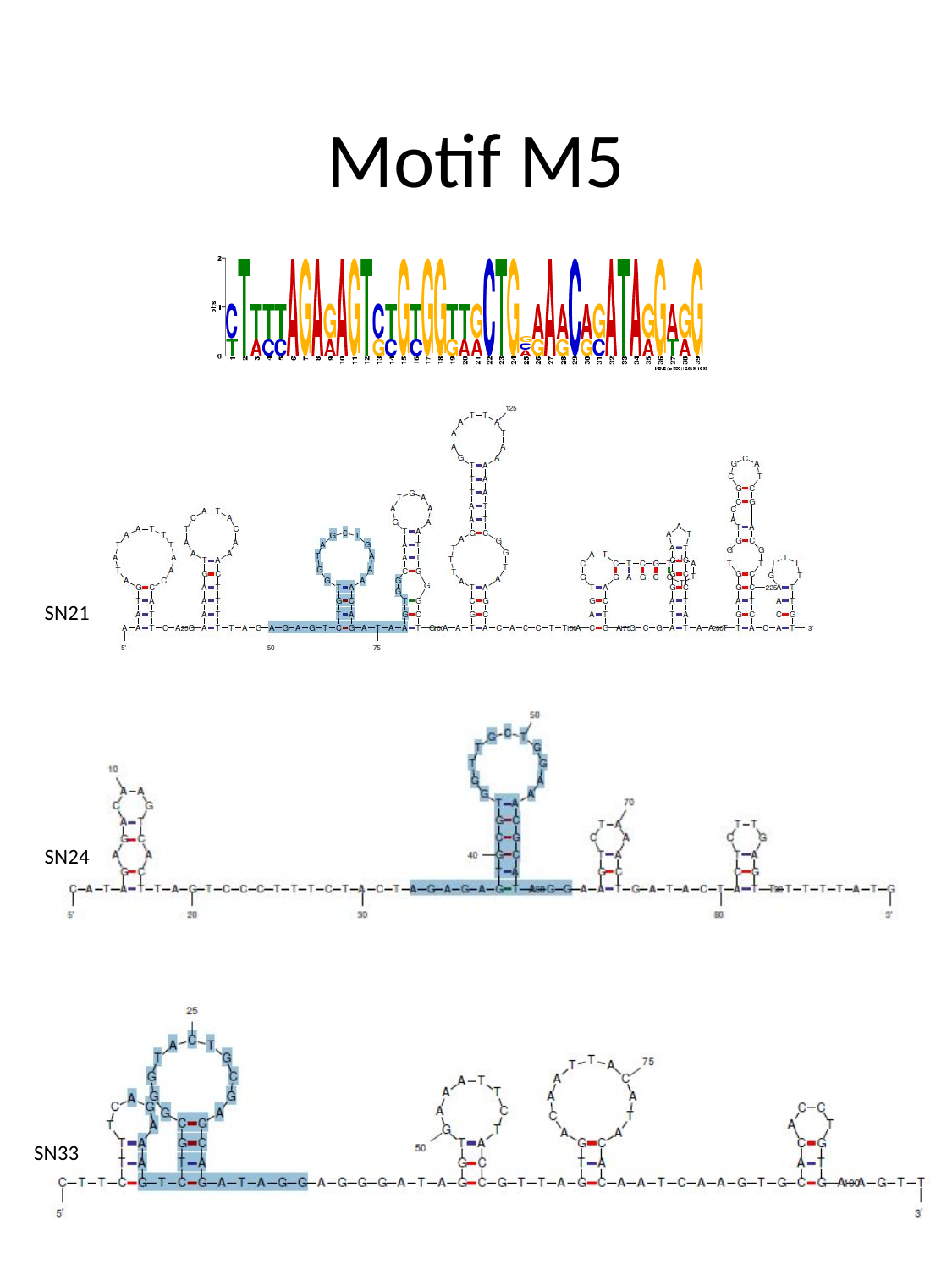

# Motif M5
SN21
SN24
SN33

## Slide 5
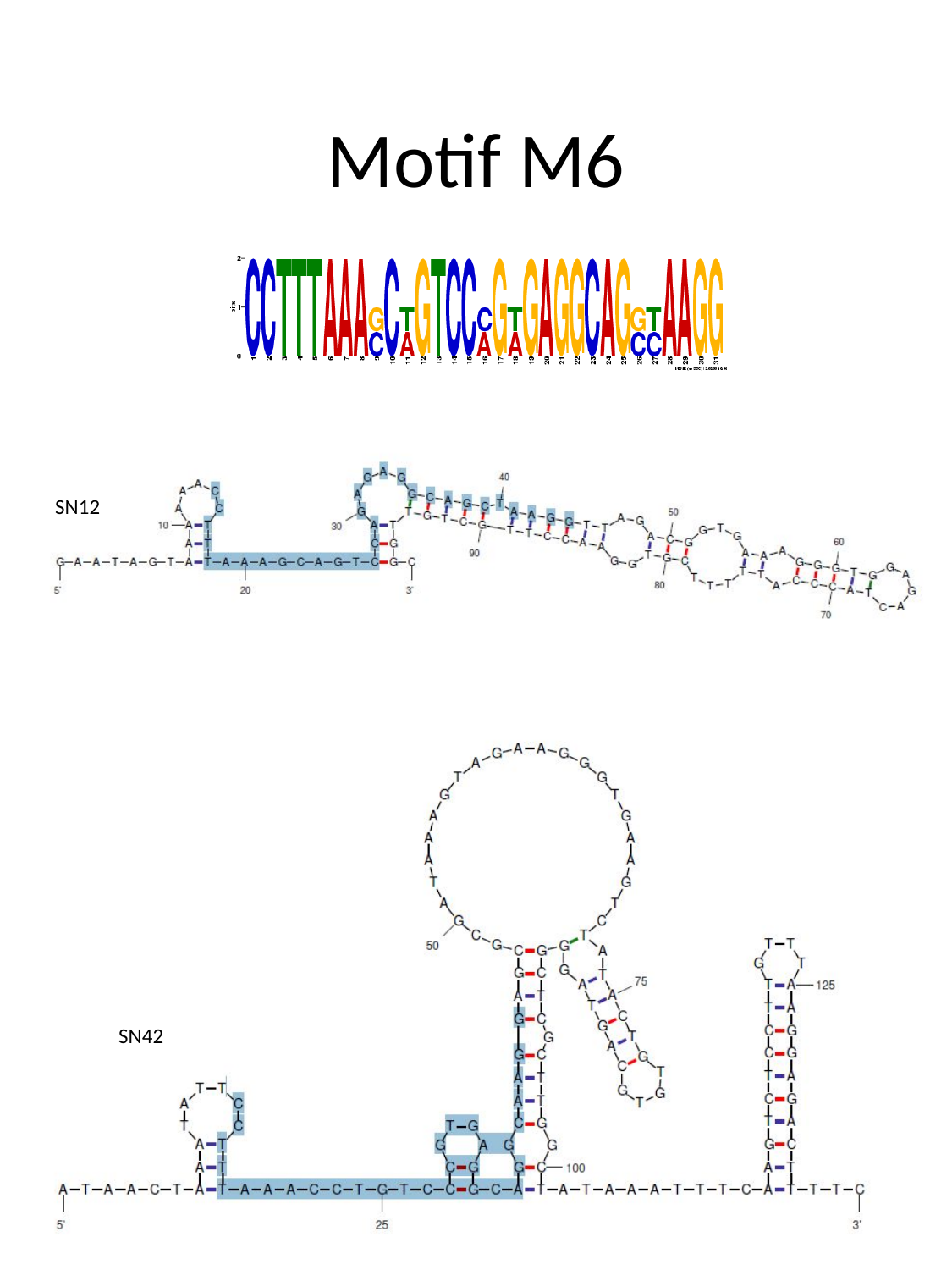

# Motif M6
SN12
SN42

## Slide 6
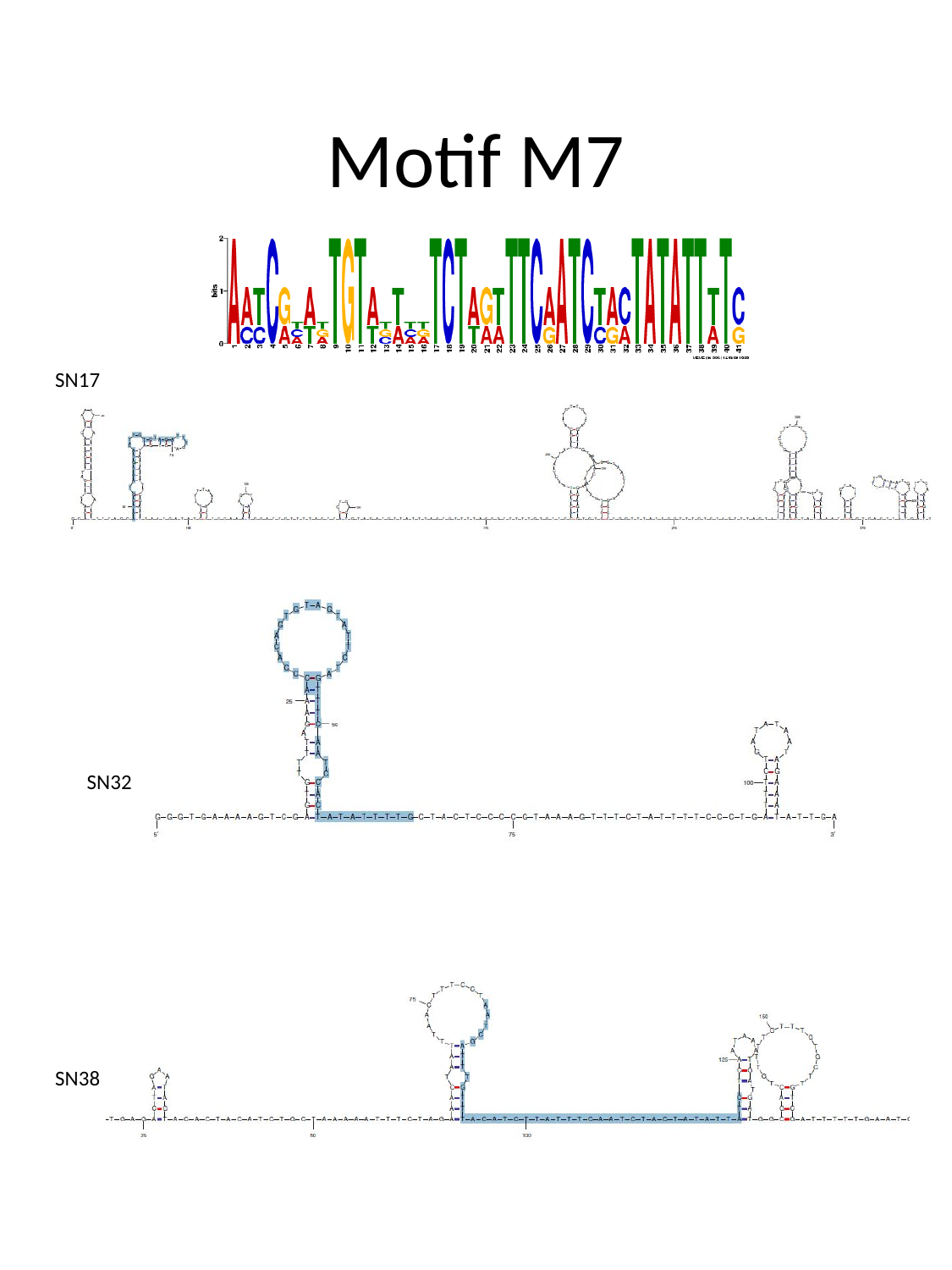

# Motif M7
SN17
SN32
SN38

## Slide 7
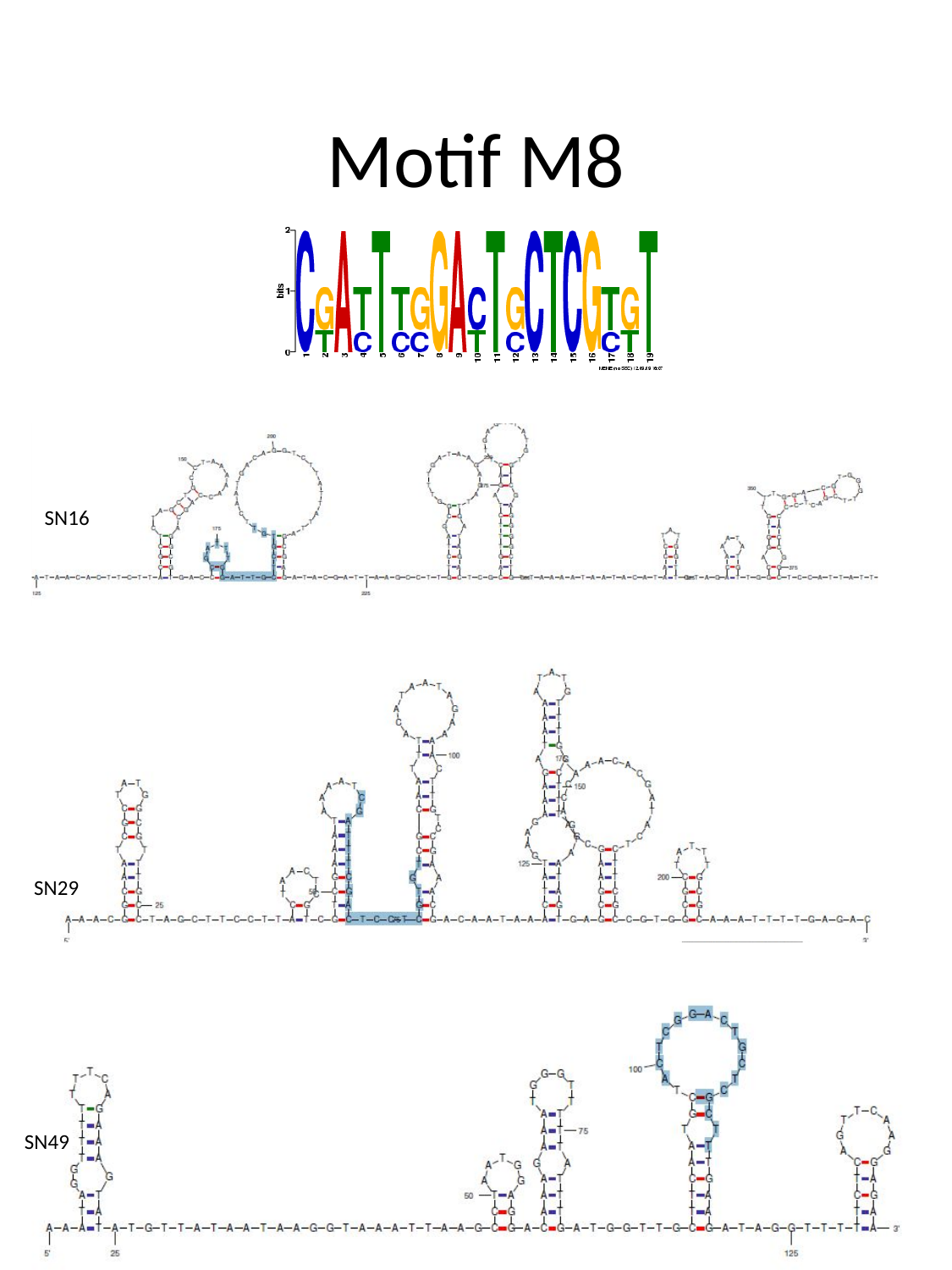

# Motif M8
SN16
SN29
SN49
