# Supplementary figures and images for: Identification of novel non-coding small RNAs from Streptococcus pneumoniae TIGR4 using high-resolution genome tiling arrays
Source: BMC Genomics. 2010 Jun 3;11:350. doi: 10.1186/1471-2164-11-350 (PMC2887815; doi:10.1186/1471-2164-11-350)

## Slide 1
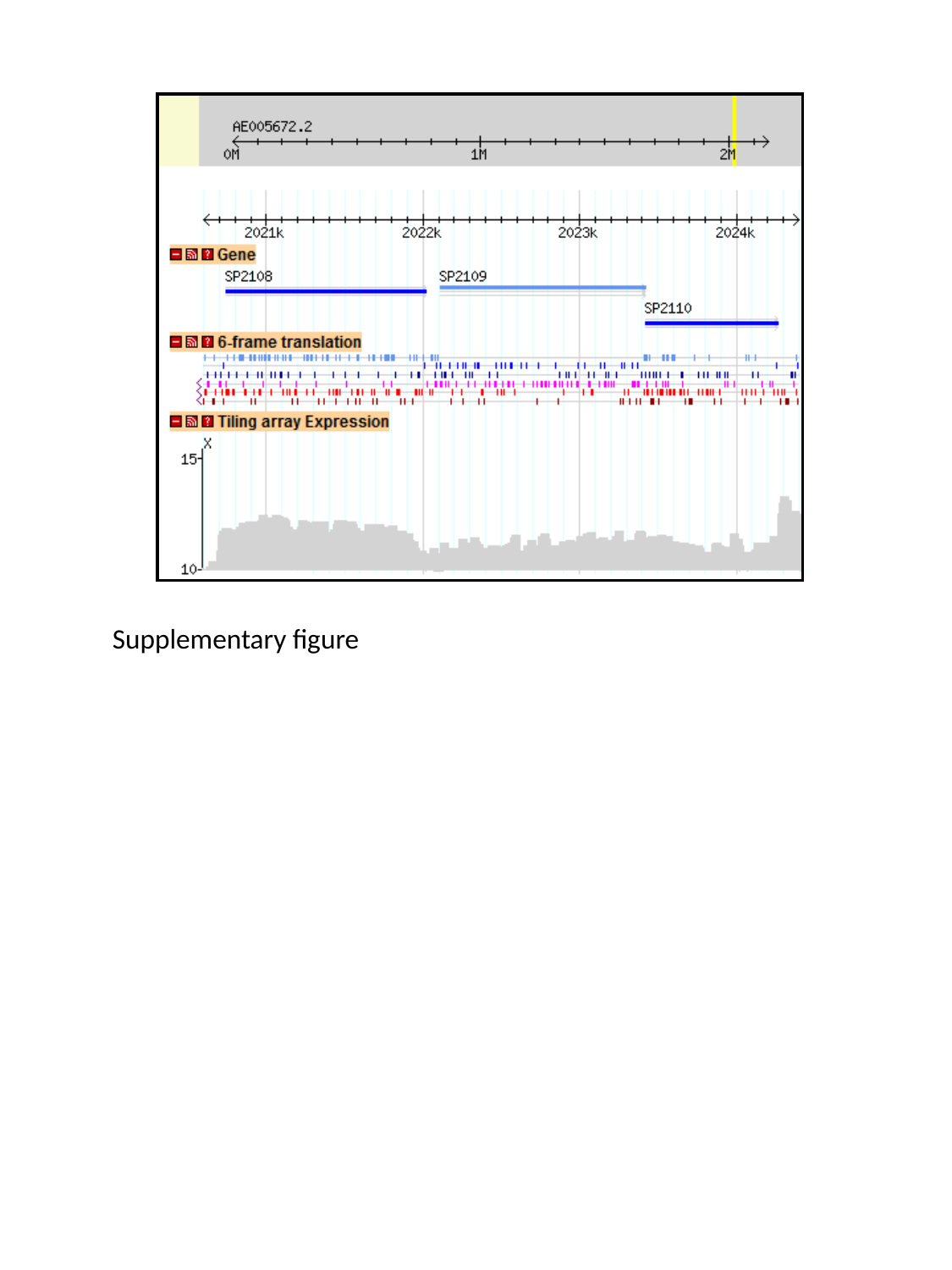

Supplementary figure

Supplement: Additional file 8 — GBrowse visualization of a transcription unit. Genome browser visualization of genes SP2108 - SP2110. The tracks shown include translation in all six frames and tiling array expression. All three genes are present in the forward strand. The "tiling array expression" track clearly shows high level of expression for SP2108 compared to SP2109-SP2110. [file 1471-2164-11-350-S8.PPT]
